# Investigating the association of health system characteristics and health care utilization: a multilevel model in China’s ageing population

**DOI:** 10.7189/jogh.10.020802

**Published:** 2020-12

**Authors:** Chaofan Li, Chengxiang Tang, Haipeng Wang

**Affiliations:** 1 Tsinghua Shenzhen International Graduate School, Tsinghua University, Shenzhen, Guangdong, China; 2 School of Public Administration, Guangzhou University, Guangzhou, Guangdong, China; 3 Centre for Health Management and Policy Research, School of Public Health, Cheeloo College of Medicine, Shandong University, Jinan, Shandong, China; 4 NHC Key Laboratory of Health Economics and Policy Research (Shandong University), Jinan, Shandong, China

## Abstract

**Background:**

To achieve universal health coverage in China, it is necessary to identify access barriers to health care. This study examined the association between health system characteristics and health care utilization in China and identified factors associated with accessing health care among the mid-aged and elderly.

**Methods:**

Data were obtained from the 2015 China Health and Retirement Longitudinal Study, and 17 370 respondents aged 45 and above were included in the analysis. The dependent variables were the use of outpatient and inpatient care among respondents. Health system characteristics at the provincial level were measured using the density of doctors and ward beds, health expenditure per visit/admission and health financing. A two-level logistic regression model was constructed to examine association between health care utilization and health system characteristics, controlling for predisposing, enabling and need variables.

**Results:**

Of the 17 370 respondents, 18.3% had utilized outpatient care and 13.7% had utilized inpatient care in 2015. Increases in the share of out-of-pocket (OOP) payments as total health spending at the provincial-level was less likely to be associated with outpatient care utilization (odds ratio (OR) = 0.96, 95% confidence interval (CI) = 0.93-0.98) among the mid-age and elderly population. Increases in the share of OOP payments (OR = 0.98, 95% CI = 0.97-1.00) and health expenditure per admission (OR = 0.20, 95% CI = 0.04-0.88) were less likely to be associated with inpatient care utilization, while increases in the density of beds (OR = 1.26, 95% CI = 1.10-1.43) was more likely to be associated with inpatient care utilization. gross domestic product (GDP) per capita at the provincial level and types of health insurance owned by respondents were significantly related to both inpatient and outpatient care utilization.

**Conclusions:**

Low affordability of the mid-aged and elderly population is the main barrier to utilizing health care in China. In order to improve access to health care, the government should make more efforts, such as improving health insurance reimbursement rates and implementing prospective provider payment methods, to decrease OOP payment for the ageing population.

Achieving equal access to health care is an important health policy goal in most countries [1-3]. According to the Sustainable Development Goals set by the United Nations in 2015, every country should take action to achieve universal health coverage (UHC), including the provision of financial risk protection and access to health care for all [4]. In China, the 2009 health care reform had set the goal to improve equitable access to basic health services for all through establishing universal health insurance coverage and reforming health care delivery system [5].

China has three social health insurance programs, including the Urban Employees’ Basic Medical Insurance (UEBMI, launched in 1997), the New Cooperative Medical Scheme (NCMS, launched in 2003), and the Urban Residents’ Basic Medical Insurance (URBMI, launched in 2007), which were established to cover all China’s citizens and to improve equal access to health care for all [5]. Since 2011, China almost achieved universal health insurance coverage [6]. However, variations exist across insurance programs in terms of their premiums, pooled level, benefit package and reimbursement rates, which creates barriers to equal access to health care [7].

Since the health care reform, China made a lot of efforts and investments to build medical infrastructure and strengthen the health workforce, and achieved great improvements in the availability of health care during the past decade. The ward beds per 1000 inhabitants has increased from 3.3 in 2009 to 5.1 in 2015, and density of health professionals increased from 4.2 to 5.8 [8].

Although China made a number of efforts to improve access to health care [9,10], inequity in health care utilization still exists across different groups with different income and education levels, as well as across regions [11,12]. Due to fast aging of populations, access to care has become a major issue for mid-aged and elderly adults in China. The older adults usually have high prevalence of non-communicable diseases, and high levels of functional impairment [13,14]. The ageing population met huge and urgent health care demands, which posed great challenge on the health care system. Therefore, it is necessary to identify barriers to equal access to health care among mid-aged and elderly adults, which could provide policy implications to improve access to care for all and achieve UHC in China.

Access to health care is a complex concept. Although some researchers defined access as freedom or opportunity to use health services [15], most viewed access as timely actual use of health services according to need [16,17]. There is consensus that access should reflect the “degree of fit” between supply- and demand-related factors and address the barriers to access from both the demand and the supply side [18,19]. To identify barriers to access from two sides empirically, in this paper, we defined access as health services utilization [20-24]. Andersen developed a behavior model of health care utilization comprising contextual and individual characteristics to identify access barriers to health care from both supply and demand perspectives [25]. Andersen’s behavior model can be used as conceptual framework to identify barriers and examine factors associated with access to health care. We attempted to apply them into a two-level analytical framework consisting of health system characteristics as high-level factors and individual characteristics as the first-level factors. The simplified analytical framework of health care utilization with health system and individual characteristics is provided in Figure S1 in the **Online Supplementary Document**.

Previous studies mainly focused on individual and household characteristics of health care utilization, highlighting the association of both individual and province-level factors with health inequity among older people in China [26]. Moreover, some studies explored regional variations in medical resource allocation and health care service provision [27]. These studies identified barriers in access to health services from individual factors. A review of China’s studies on health care utilization studies found that empirical research in China paid greater attention to individual factors rather than contextual factors [28]. To our knowledge, only a few studies focused on the association between contextual factors and health care utilization in China. For example, Huang and Fan studied the correlation between contextual factors (e.g., region of residence and community characteristics) and preventative care utilization [29,30], and Jin found that higher density of health workforce was associated with improved health care accessibility [31]. However, little empirical research has examined the extent to which the health system characteristics are associated with health care utilization among the mid-aged and elderly in China. Therefore, a close examination of the association of health system characteristics and health care utilization in China is imperative.

This paper examined the association of health system characteristics and health care utilization among the mid-aged and elderly, and identified the main barriers in accessing health services from both the supply and demand sides.

## METHODS

### Selection and description of participants

This study used a cross-sectional study design to examine the association between health system characteristics and health care utilization. Data were obtained from the fourth wave of the China Health and Retirement Longitudinal Study (CHARLS) that provided a nationally representative sample of mid-aged and elderly residents in China (http://charls.pku.edu.cn/index.html). Residents aged 45 and older were selected from 150 counties within 28 provinces (excluding Ningxia, Hainan, and Tibet) through multistage probability-proportional-to-size sampling methods. Participants were interviewed by a trained interviewer using face-to-face computer-assisted personal interviews and standardized questionnaires. More details on CHARLS can be found in the cohort profile, and data are publicly available at the website of the Institute of Social Science Survey, Peking University [32]. In 2015, 12 221 households and 19 840 family members aged 45 years or older were interviewed, with a response rate of 82.1%. The inclusion criteria were: (1) aged 45 and above; (2) community-dwelling. Observations with missing values in key outcome and independent variables were excluded. The final sample size included in this study was 10 923 households and 17 370 respondents. Data on health system characteristics were obtained from the Statistical Yearbook of Health and Family Planning of China (2016), which included information on gross domestic product (GDP) per capita, health resource allocation, and health financing and expenditures at the provincial levels [8].

### Measurement

Health care utilization refers to the use of services by persons to prevent and cure health problems, promote maintenance of health and well-being [33]. It could be broadly measured as, for example, whether or not a person was hospitalized or used outpatient care, emergency department and dental care or the frequency of these visits [34]. Van Doorslaer found that whether a person used health care rather than the number of visits that person occurred contribute to inequality in health care utilization [35]. Thus, we used dummy variables indicating whether or not the respondents used outpatient and inpatient care utilization, respectively, as the outcome variables in this study. Use of outpatient care was measured by asking respondents whether they visited the outpatient departments in a health institution or a physician office in the last month, while use of inpatient care was measured by asking respondents whether they received inpatient care in the past year. Both outcome variables were coded as dichotomous, with 0 = no and 1 = yes.

We measured the health system characteristics (including health financing and availability of health resources) using provincial-level data, because data at the county- and community-level are not available. Health care financing relates to people’s affordability of health care. Consistent with previous studies, we measured health financing using three variables, including per capita GDP, per capita expenditure per visit or admission, and the share of out-of-pocket (OOP) payment of total health expenditure (THE) [25,36,37]. Per capita GDP varies across provinces in China. High health care expenditure per visit or admission and OOP payment share creates financial barriers to accessing health care.

The density of physicians and ward beds were used to measure the availability of health resources that determines the supply of health services. Based on previous studies and the statistical indicator used by the National Commission of Health in China, we used the number of health professionals and ward beds per 1000 residents to measure health resource availability [17,38].

Individual-level characteristics included predisposing, health care needs, and enabling variables. Demographic characteristics, marital status, education, and occupation were selected as predisposing factors. Demographic characteristics included gender (female, male) and age groups (45-59, 60-74, 75 and above). Marital status was classified into two groups: (1) married and partnered, and (2) widowed, divorced, and others. Education was coded into four groups: (1) lower than primary school, (2) primary school, (3) middle school, and (4) high school and above. Occupation status was categorized into four groups: (1) agricultural work, (2) employed, (3) self-employed, and (4) unemployed and retired.

Enabling factors included types of health insurance coverage, areas of residence, and household income level. Health insurance coverage type was grouped into: (1) uninsured, (2) NCMS, (3) URBMI, (4) UEBMI, and (5) other health insurance schemes. Areas of residence were urban community and rural village areas. Household income level was grouped into quantiles based on the rank of per capita household consumption expenditure. Household consumption had proved to be a good measure for living standard in a prior study that also used CHARLS data [39].

Health care need variables included self-reported health status and diagnosis with chronic disease. Respondents were asked to rate their overall health status on a 5-point scale, including excellent, very good, good, fair, and poor, and was recoded into three categories: good, fair, and poor in the analysis. Fourteen categories of chronic disease were identified in the CHARLS survey, such as diabetes, hypertension, and chronic lung diseases. If a respondent reported that he or she had been diagnosed with any kind of chronic disease, he or she was considered being a person diagnosed with chronic disease, otherwise not.

### Statistical analysis

We constructed a multi-level logistic regression model to examine the association of health care utilization between health system characteristics and individual characteristics. Following the procedure of developing a multilevel model suggested by Merlo and Sommet [40,41], we first ran a two-level logistic model with no individual- and provincial-level variables (null model):


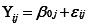
(1)


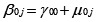
(2)

in which Y_ij_ is the dummy outcome variable (*i* refers to the individual and *j* refers to the province), β_0j_ is the mean of probability of health care utilization in province *j*, ϵ_ij_ is the residual component for individual *i* in province *j*, γ_00_ is the mean value of outcome variable across all provinces, and μ_0j_ denotes the deviation of province *j*’s mean from the grand mean of all individuals. μ_0j_ usually refers to random intercept effect, which is assumed to have a mean of 0 and constant variance σ^2^. Intraclass correlation (ICC) was calculated to assess the degree of variation that could be attributed to contextual level variables [42]:


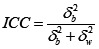
(3)

in which σ_b_^2^ represents the between-province variance and σ_w_^2^ denotes the within-province variance. When the ICC is significantly different from zero, within-cluster correlation exists and one could consider running multilevel regression analysis [41]. Then, we included individual covariates to the first-level equation and fitted a random intercept model (Model 1):


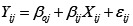
(4)


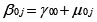
(2)

in which X_ij_ is the vector of individual predisposing, enabling, and need variables, and β_i_ denotes the vector of their coefficients. Lastly, we introduced the health system characteristics of each province into level-2 equations, controlling for individual factors to distinguish the association of health system characteristics and health care utilization (Model 2):


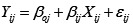
(4)


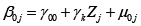
(5)

in which Z_j_ is the vector of health system factors, and γ_k_ denotes the vector of their coefficients. The odds ratio (OR) and 95% confidence interval (CI) of covariates were estimated to represent the impact of individual characteristics and health system characteristics on health care utilization. Akaike information criterion, Bayesian information criterion and likelihood ratio test were used to compare the goodness of fit of different models [43]. All statistical analyses were conducted using the software Stata 15.0. (StataCorp LLC, College Station TX, USA).

### Ethics considerations

This study is exempt because it used secondary data from the publicly available data source (http://charls.pku.edu.cn/index.html). The CHARLS was obtained ethical approval from the Ethical Review Committee at Peking University in January 2011.

## RESULTS

### Health care utilization and basic characteristics of respondents

**Figure 1:**
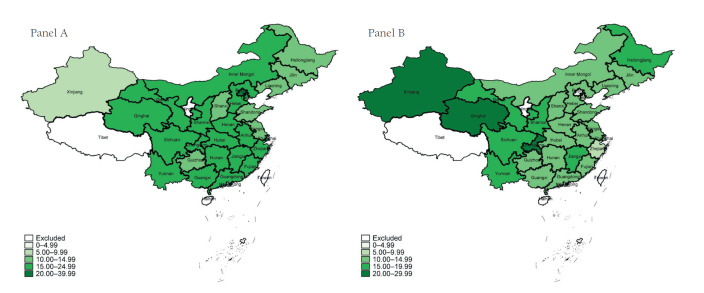
Probability of outpatient and inpatient care utilization among mid-aged and elderly in China in 2015. **Panel A.** Probability of outpatient care utilization. **Panel B.** Probability of inpatient care utilization.

Descriptive statistics of health care utilization and respondents’ characteristics are presented in **Table 1**. More than 18% of the mid-aged and elderly respondents had used outpatient care during the past month and about 14% had used hospitalization services during the last year. There were significant variations in the use of outpatient and inpatient care utilization among mid-aged and elderly adults across provinces (**Figure 1**). The percent of outpatient visits during the last month ranged from 5% to 38% (**Figure 1**, Panel A), and the percent of inpatient visits during the last year varied from 4% to 27% (**Figure 1**, Panel B).

**Table 1:** Descriptive characteristics of respondents

Variables	Categories	N	%
Outpatient care utilization	No	14 187	81.68
	Yes	3183	18.32
Inpatient care utilization	No	14 987	86.28
	Yes	2383	13.72
Gender	Female	8854	50.97
	Male	8516	49.03
Age	45-59	8779	50.54
	60-74	7137	41.09
	75+	1454	8.37
Marital status	Married and partnered	15 181	87.40
	Widowed, divorced and others	2189	12.60
Education	Lower than primary school	7188	41.38
	Primary school	4766	27.44
	Middle school	3470	19.98
	High school and above	1946	11.20
Occupation status	Agricultural work	7302	42.04
	Employed	3192	18.38
	Self-employed	1503	8.65
	Unemployed and retired	5373	30.93
Self-reported health status	Good	4352	25.05
	Fair	9131	52.57
	Poor	3887	22.38
Chronic disease	No	5140	29.59
	Yes	12 230	70.41
Health insurance coverage	Uninsured	1315	7.57
	NCMS	11 725	67.50
	URBMI	1184	6.82
	UEBMI	2024	11.65
	Others	1122	6.46
Region of residence	Urban community	6733	38.76
	Rural village	10 637	61.24
Total		17 370	100.00

NCMS – New Cooperative Medical Schemes; URBMI – Urban Residents’ Basic Medical Insurance; UEBMI – Urban Employees’ Basic Medical Insurance.

The majority (87%) of residents were married or partnered, 41% were low-educated, 42% were engaged in agricultural work, 31% were not working. More than half (53%) reported fair health status and 70% were diagnosed with at least one kind of chronic disease. More than 22% rated their health status as poor. Almost all respondents (92%) had health insurance coverage, so only 8% were uninsured. More than 60% of respondents were living in rural villages, compared to 39% living in urban communities.

### Health system characteristic at the provincial level

**Figure 2:**
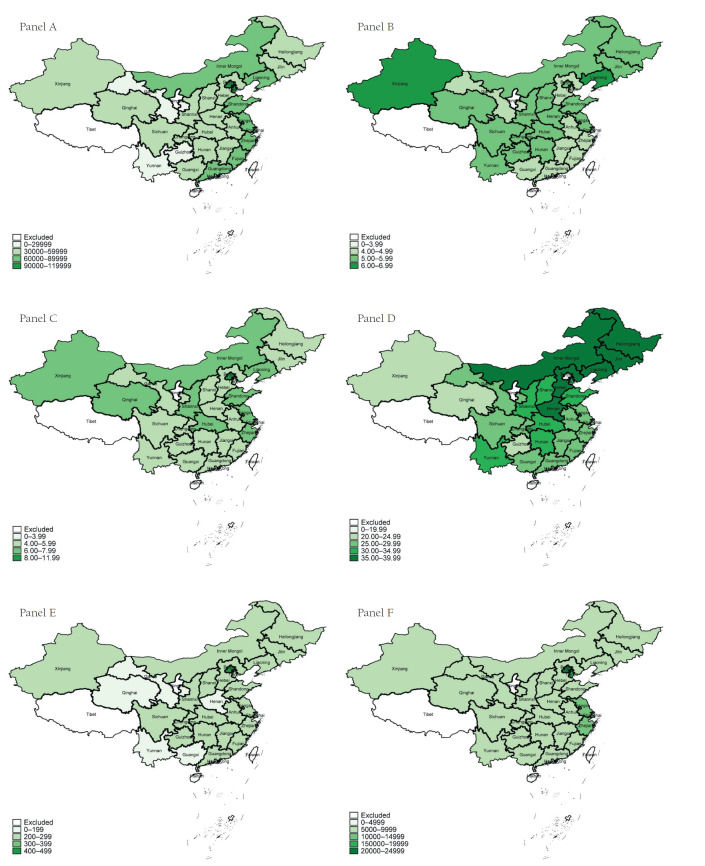
Economic development and health system characteristics of 28 provinces in China in 2015. **Panel A.** Gross Domestic Product Per capita (Chinese yuan). **Panel B.** Ward Beds per 1000 inhabitant. **Panel C.** Health professionals per 1000 inhabitants. **Panel D.** Share of out-of-pocket payment in total health expenditure (%). **Panel E.** Outpatient expenditure per visits (Chinese yuan). **Panel F.** Inpatient expenditure per admission (Chinese yuan).

**Figure 2** and Table S1 in the **Online Supplementary Document** show the economic development and several health system characteristics at the provincial level in China in 2015. Economic development varied greatly across provinces (**Figure 2**, Panel A), for example, per capita GDP in Beijing (CNY¥106497, US$17099) was 3.13 times higher than that in Gansu (CNY¥26165, US$4201). There were also large variations in health resources allocation across provinces. The number of ward beds per 1000 residents ranged from 4.02 to 6.37 (**Figure 2**, Panel B), while the number of health professionals per 1000 residents ranged from 4.6 to 10.4 (**Figure 2**, Panel C). Citizens in developed regions may also receive better financial protection for health services (**Figure 2**, Panel D). The share of OOP payment as THE in Beijing (17.39%) was much lower than that in other provinces (around 30%). Health expenditure per visit or admission also varied widely across provinces (**Figure 2**, Panels E and F), for example outpatient expenditure per visit in Beijing was 2.68 times as high as that in Henan province (CNY¥440.90 vs 164.40, US$70.79 vs 26.40), and inpatient expenditure per admission in Beijing was 3.74 times as high as that in Guizhou province (CNY¥20149.20 vs 5387.70, US$ 3235.05 vs 865.02).

### Health system characteristics and individual factors associated with health care utilization

**Table 2** reports the results from the multilevel logistic regression testing the association of outpatient care utilization with health system and individual characteristics. Compared to female respondents, male respondents were 0.80 (95% CI = 0.73-0.87) times less likely to seek outpatient care. Respondents who received high school and above education (OR = 1.24, 95% CI = 1.06-1.45) were more likely to seek outpatient care than those with low educational levels. The employed older adults had a lower likelihood of visiting physicians than respondents engaging in agricultural work (OR = 0.88, 95% CI = 0.77-1.00). After adjusting for predisposing factors, need factors were still associated with outpatient care utilization. Respondents who reported fair (OR = 1.76, 95% CI = 1.56-1.98) and poor health status (OR = 3.31, 95% CI = 2.90-3.78), and respondents diagnosed with chronic disease (OR = 1.90, 95% CI = 1.70-2.12) were more likely to use outpatient care than their counterparts. Moreover, health insurance coverage, residence in a rural village, and high economic status statistically were also positively associated with outpatient care utilization.

**Table 2:** Association of health system characteristics and outpatient care utilization: multilevel regression model

Variables	Categories	Null model	Model 1	Model 2
**OR (95% CI)**	**OR (95% CI)**	**OR (95% CI)**
**FIXED EFFECTS**				
Individual level:				
-Predisposing factors				
Gender (ref = Female)	Male		0.80 (0.73, 0.87)‡	0.80 (0.73, 0.87)‡
Age (ref = 45-59)	60-74		1.01 (0.92, 1.11)	1.01 (0.92, 1.11)
	75+		0.91 (0.76, 1.07)	0.91 (0.76, 1.07)
Marital status (ref = Married and partnered)	Widowed, divorced and others		1.02 (0.90, 1.15)	1.02 (0.90, 1.15)
Education (ref = Lower than primary school)	Primary school		1.07 (0.97, 1.19)	1.07 (0.97, 1.19)
	Middle school		1.09 (0.97, 1.23)	1.09 (0.97, 1.23)
	High school and above		1.24 (1.06, 1.45)‡	1.24 (1.06, 1.45)‡
Occupation status (ref = Agricultural work)	Employed		0.88 (0.77, 1.00)*	0.88 (0.77, 1.00)†
	Self-employed		0.92 (0.78, 1.08)	0.92 (0.78, 1.08)
	Unemployed and retired		0.93 (0.84, 1.03)	0.93 (0.84, 1.03)
Need factors				
Self-reported health status (ref = Good)	Fair		1.75 (1.55, 1.98)‡	1.76 (1.56, 1.98)‡
	Poor		3.30 (2.89, 3.77)‡	3.31 (2.90, 3.78)‡
Chorionic disease (ref = No)	Yes		1.90 (1.70, 2.12)‡	1.90 (1.70, 2.12)‡
Enabling factors				
Health insurance (ref = Uninsured)	NCMS		1.37 (1.16, 1.62)‡	1.37 (1.16, 1.62)‡
	URBMI		1.42 (1.14, 1.78)‡	1.42 (1.14, 1.78)‡
	UEBMI		1.57 (1.28, 1.94)‡	1.57 (1.28, 1.94)‡
	Others		1.46 (1.16, 1.84)‡	1.46 (1.16, 1.84)‡
Region of residence (ref = urban community)	Rural village		1.13 (1.03, 1.25)‡	1.13 (1.03, 1.25)‡
Economic status (ref = Poorest)	2		1.11 (0.97, 1.26)	1.11 (0.97, 1.26)
	3		1.13 (0.99, 1.28)*	1.13 (0.99, 1.28)*
	4		1.10 (0.96, 1.25)	1.10 (0.96, 1.25)
	Richest		1.26 (1.10 1.43)‡	1.25 (1.09, 1.42)‡
Constant		0.22 (0.19, 0.25) ‡	0.05 (0.04, 0.06) ‡	0.01 (0.00, 0.96)
Provincial level				
Per capita GDP				1.57 (1.03, 2.40)†
Share of OOP in THE				0.96 (0.93, 0.98)‡
Log of outpatient expenditure per visit				0.62 (0.08, 5.09)
Number of health professionals per 1000 inhabitants				0.82 (0.53, 1.25)
**RANDOM EFFECTS**				
Level-2 intercept variance			0.13 (0.07, 0.27)	0.07 (0.03, 0.15)
Number of observations		17 370	17 370	17 370
AIC		16 412.79	15 637.34	15 630.96
BIC		16 428.31	15 823.64	15 848.31
Intraclass correlation		0.04	0.04	0.02
Log likelihood		-8204.39	-7794.67	-7787.48
LR test statistic		*P* < 0.001	*P* < 0.001	*P* < 0.001

OR – odds ratio; CI – confidence interval; NCMS – New Cooperative Medical Schemes; URBMI – Urban Residents’ Basic Medical Insurance; UEBMI – Urban Employees’ Basic Medical Insurance; GDP – Gross Domestic Product; OOP - out-of-pocket; THE – total health expenditure; AIC – Akaike information criterion; BIC – Bayesian information criterion; LR test – likelihood ratio test

*, *P* < 0.1; †, *P* < 0.05; ‡, *P* < 0.01.

The random intercept variance at the provincial level indicated that the likelihood of outpatient visits varied across provinces, even after controlling for individual-level factors. Model 2 shows that increases in per capita GDP were positively associated with outpatient care (OR = 1.57, 95% CI = 1.03-2.40). In contrast, increases in OOP payment share were negatively associated with outpatient care utilization (OR = 0.96, 95% CI = 0.93-0.98). Neither outpatient expenditure per visit nor the number of health professionals per 1000 residents was significantly associated with outpatient service.

**Table 3** presents the regression results testing the relationship between provincial health system features and individual-level factors and inpatient care utilization. Compared to the reference groups, male (OR = 1.11, 95% CI = 1.01-1.23), those aged 60-74 (OR = 1.25, 95% CI = 1.12-1.39), those aged 75 years and above (OR = 1.43, 95% CI = 1.20-1.70), and those unemployed or retired (OR = 1.61, 95% CI = 1.44-1.81) were more likely to seek inpatient care, while respondents received high school and above education (OR = 0.78, 95% CI = 0.65-0.94) and the employed (OR = 0.83, 95% CI = 0.71-0.98) were less likely to seek inpatient services. After adjusting for predisposing factors, need factors were the major factors associated with outpatient care utilization. Fair health status (OR = 1.67, 95% CI = 1.44-1.93), poor health status (OR = 4.16, 95% CI = 3.57-4.86), and presence of chronic disease (OR = 2.31, 95% CI = 2.00-2.65) were significantly associated with inpatient care utilization. Furthermore, after adjusting for predisposing and enabling factors, there were statistically significant associations between enabling factors and use of inpatient services. There was a strong gradient in the relationship between economic status and hospitalization. Moreover, compared to the uninsured, respondents covered by health insurance were more likely to utilize inpatient services.

**Table 3:** Association of health system characteristics and inpatient care utilization: multilevel regression model

Variables	Categories	Null model	Model 3	Model 4
**OR (95% CI)**	**OR (95% CI)**	**OR (95% CI)**
**FIXED EFFECTS**				
**Individual level:**				
Predisposing factors				
Gender (ref = Female)	Male		1.11 (1.01, 1.23)†	1.11 (1.01, 1.23)†
Age (ref = 45–59)	60-74		1.24 (1.11, 1.38)‡	1.25 (1.12, 1.39)‡
	75+		1.42 (1.19, 1.69)‡	1.43 (1.20, 1.70)‡
Marital status (ref = Married and partnered)	Widowed, divorced and others		1.03 (0.90, 1.18)	1.03 (0.90, 1.17)
Education (ref = Lower than primary school)	Primary school		0.96 (0.85, 1.08)	0.96 (0.85, 1.08)
	Middle school		0.94 (0.82, 1.08)	0.94 (0.82, 1.08)
	High school and above		0.78 (0.65, 0.94)‡	0.78 (0.65, 0.94)‡
Occupation status (ref = Agricultural work)	Employed		0.82 (0.69, 0.96)†	0.83 (0.71, 0.98)†
	Self-employed		1.18 (0.98, 1.43)*	1.19 (0.99, 1.43)*
	Unemployed and retired		1.59 (1.41, 1.79)‡	1.61 (1.44, 1.81)‡
**Need factors:**				
Self-reported health status (ref = Good)	Fair		1.67 (1.44, 1.94)‡	1.67 (1.44, 1.93)‡
	Poor		4.16 (3.57, 4.86)‡	4.16 (3.57, 4.86)‡
Chorionic disease (ref = No)	Yes		2.32 (2.01, 2.67)‡	2.31 (2.00, 2.65)‡
Enabling factors				
Health insurance (ref = Uninsured)	NCMS		1.43 (1.18, 1.74)‡	1.42 (1.17, 1.73)‡
	URBMI		1.66 (1.29, 2.13)‡	1.64 (1.28, 2.10)‡
	UEBMI		1.69 (1.34, 2.13)‡	1.68 (1.33, 2.13)‡
	Others		1.58 (1.22, 2.05)‡	1.60 (1.24, 2.08)‡
Region of residence (ref = Urban community)	Rural village		1.02 (0.91, 1.14)	1.00 (0.89, 1.11)
Economic status (ref = Poorest)	2		1.18 (1.00, 1.38)†	1.18 (1.00, 1.38)†
	3		1.48 (1.27, 1.73)‡	1.48 (1.27, 1.73)‡
	4		1.76 (1.51, 2.05)‡	1.76 (1.52, 2.05)‡
	Richest		2.19 (1.88, 2.55)‡	2.19 (1.88, 2.56)‡
Constant		0.16 (0.14, 0.18)	0.01 (0.01, 0.02)‡	5.68 (0.21, 151.84)‡
**Provincial level:**				
Per capita GDP				0.99 (0.69, 1.42)
Share of OOP in THE				0.98 (0.97, 1.00) †
Log of inpatient expenditure per admission				0.20 (0.04, 0.88) †
Ward Beds per 1000 inhabitants				1.21 (1.09, 1.35)‡
**RANDOM EFFECTS**				
Level-2 intercept variance		0.05 (0.02, 0.12)		0.01 (0.00, 0.04)
Number of observations		17 370	17 370	17 370
AIC		13 838.93	12 483.55	12 461.71
BIC		13 854.45	12 669.85	12 679.06
Intraclass correlation		0.02	0.01	0.002
Log likelihood		-6917.46	-6217.77	-6202.86
LR test statistic		*P* < 0.001	*P* < 0.001	*P* = 0.07

OR – odds ratio, CI – confidence interval, NCMS – New Cooperative Medical Schemes, URBMI – Urban Residents’ Basic Medical Insurance, UEBMI – Urban Employees’ Basic Medical Insurance, GDP – Gross Domestic Product, OOP – out-of-pocket, THE – total health expenditure, AIC – Akaike information criterion, BIC – Bayesian information criterion, LR test – likelihood ratio test

*P < 0.1.

†*P* < 0.05

‡*P* < 0.01.

Similar to outpatient care utilization, the likelihood of inpatient service utilization varied across provinces, even after adjusting for individual-level factors. Model 4 shows that OOP payment share was significantly negatively associated with inpatient care utilization (OR = 0.98, 95% CI = 0.97-1.00). High health expenditure per admission was also negatively associated with hospitalization (OR = 0.20, 95% CI = 0.04-0.88). In contrast, the number of ward beds per 1000 residents was significantly positively associated with inpatient care use (OR = 1.21, 95% CI = 1.09-1.35). Lastly, no significant association was observed between per capita GDP and inpatient care utilization.

## DISCUSSION

To identify the main barriers of access to health care in the ageing population in China, this study analyzed the association of provincial health system characteristics and health care utilization using a multilevel regression model based on nationally representative survey data. We have two main important findings. The first is that large variation exists across provinces in China in terms of the density of health resources, health service expenditure per visit/admission, and the share of OOP payment as THE, reflecting the disparity in health care availability and affordability across China. The second important finding is that affordability of health care is the major barrier to health care utilization among the mid-aged and elderly in China. A high OOP payment share was negatively associated with outpatient and inpatient care utilization, and health expenditure per admission was negatively associated with use of inpatient care. These findings added to the existing evidence on the association between health system characteristics and health care utilization among the mid-aged and elderly in China.

### Variation in health system characteristics across provinces in China

Although the Chinese government has improved the density of ward beds and physicians and decreased user fees for health care services, the disparity in availability and affordability still remains across provinces. During the past ten years, both the number of ward beds and health professionals per 1000 inhabitants increased rapidly [44]. Compared to Organization for Economic Co-operation and Development (OECD) countries, in 2015, China had a higher density of ward beds but a lower density of physicians [45]. Moreover, OOP payments accounted for one-third of THE in China, much higher than that in OECD countries in 2015 [45]. Furthermore, the differences between the highest and lowest values of each health system characteristics across 28 provinces indicates that large variation exists in the density of ward beds and health professionals, health service expenditure per capita, OOP payment share of THE, and health care utilization. Previous cross-sectional studies have revealed disparities in health resource allocations across regions [46-49]. Prior trend analyses also showed that disparity in ward beds narrowed, while pro-rich inequality among health professionals remained [44,50].

### Bed density associated with inpatient care utilization

Our results suggest that high availability of ward beds could be positively related with inpatient care utilization among the mid-aged and elderly. This confirms Roemers’ hypothesis: an increase in the number of ward beds per capita always increases the hospital utilization rate [51]. Furthermore, a previous study conducted in China suggested that the number of ward beds available is positively associated with the frequency of hospitalization [52]. A plausible reason for the Roemer effect is that high supply of ward beds may induce demands for hospital services. In theory, hospitals face financial incentives to provide additional care to patients when beds are available [53]. An increase in ward bed supply is usually accompanied by an increase in hospital care utilization [54].

### Financial affordability associated with health care utilization

Financial affordability is still a major barrier to universal access to health care in China. The result that the share of OOP payment of THE and per capita health expenditure were negatively associated with health care utilization is consistent with previous studies in other countries [1,55]. It is widely known that universal health care coverage is more likely to be achieved in countries with a lower proportion of OOP payment financing [2,56]. Conversely, as the OOP payment share and health care prices increase, the financial burden also increases substantially. This study also confirmed that a higher share of OOP payment was negatively correlated with health care utilization in China. Although the OOP payment share of THE has decreased in recent years, user fees were still an important source of health financing – as high as about 30%. Furthermore, variations in reimbursement rates across different health insurance programs could exacerbate the gaps in OOP spending, which deepens disparities in access to health care. This highlights the importance of adjusting the coinsurance rates of enrollees and consolidating fragmented health insurance schemes to achieve UHC and promote equal access to health care.

More importantly, we found that inpatient expenditure per admission was negatively associated with probability of hospitalization, while outpatient expenditure per visit had no significant relationship with outpatient care utilization. High costs of services are major barriers to health care utilization in both developed and developing countries [16]. However, during the past 20 years, health care costs in China have increased rapidly because of fee-for-service payment and technological innovation [6,57,58]. Cost escalation, which obstructs the population’s access to health care, is a major concern of the public and the government. It is likely that the price elasticity of demand for outpatient care was low in China, so we did not observe a significant association between outpatient prince and outpatient utilization [59]. The negative correlation between health care price and health care utilization suggests that efforts should be taken to control cost increases and improve patients’ affordability for health care services [60,61].

### Policy implications

Our study provides important policy implications for improving equitable access to health care in China. First, China should balance the distribution of health resources. Large variations in the density of warn beds and health professionals exist between high- and low-economic development provinces. The government should develop need-based health resources allocation planning rather than historical increment list method and economic development level in order to balance the distribution of health resources. Second, China should reform health insurance system to decrease high OOP. On one hand, the government should reform providers’ payment methods (especially Diagnosis-related groups prospective payment method for acute inpatient services) to control the increase in health care expenditure. On the other hand, the government should increase the reimbursement rates of inpatient services for enrollees of URRBMI and narrow the gaps between UEBMI and URRBMI. Third, China should focus on the unenrolled population. Compared to the enrollees of social health insurance schemes, the uninsured are less likely to use outpatient and inpatient care when they need it. Therefore, strategies, including simplifying enrolment procedure and improving premium collection approaches, should be taken to expand health insurance coverage in the vulnerable groups [62].

UHC is an important global dimension [63]. However, most of low-and middle-income countries (LMIC) have not achieved UHC [64]. Moreover, older adults usually have greater need and require different approaches to health care, but are often less able to pay for services [65]. Population aging is a critical challenge to achiever UHC for LMIC [66]. The findings of this study distinguished main barriers to universal access to health care among the mid-aged and elderly from supply-side. This may have implications on how to realign health financing and resources to meet for UHC targets for LMIC.

### Limitations

This study has several limitations. First, our analysis used a cross-sectional study design and the results cannot infer causality. Future studies on causal pathways are needed to reveal how health system characteristics influence health care utilization. Second, the measurement of contextual variables was conducted at the provincial level, because data on the county level were not available through the published health statistical yearbook. This may lead to underestimating the associations of health system characteristics and health care utilization. Health system characteristics should be measured at the county level or community level in future studies to capture the within-cluster correlation and contextual effects. Third, the sample size in six provinces was lower than 300, which may impact the representativeness of samples at provincial level. This may also lead to underestimating the associations of health system characteristics and health care utilization. Forth, the CHARLS data were mainly self-reported, which may create self-report bias in presence of chronic disease and recall bias in household consumption expenditure.

## CONCLUSIONS

The study suggests that large gaps in availability of health resources, health care price and affordability exist across provinces in China. The share of OOP payment as THE and the price of health care play an important role in health care utilization among aging population in China. High OOP and health care price are major financial barriers to accessing health care among the mid-aged and elderly. To improve equal access to health care among the mid-aged elderly, China should allocate health resource according to need-based planning. Most importantly, China should take more priority measures to eliminate the affordability barriers in next phase of health system reforms, such as decreasing the coinsurance rate to reduce the OOP payment share, reforming provider payment methods to control increments in health care prices, and expanding health insurance coverage in vulnerable groups.

## Additional material

Online Supplementary Document
