# Supplementary material for: Investigating the association of health system characteristics and health care utilization: a multilevel model in China’s ageing population
Source: J Glob Health. 2020 Dec 6;10(2):020802. doi: 10.7189/jogh.10.020802 (PMC7719298; doi:10.7189/jogh.10.020802)
Supplement: Online Supplementary Document [file jogh-10-020802-s001.pdf]

Online Supplementary Documents

Table S1 Health system characteristics of 28 provinces in China in 2015

**Provinces**	***Sample size***	**Per capita GDP, CNY (US$)**	**OOP/THE**	**Outpatient expenditure per visits, CNY (US$)**	**Inpatient expenditure per admission, CNY (US$)**	**Ward Beds per 1000 inhabitants**	**Health professionals per 1000 inhabitants**
Beijing	47	106497 (17099)	17.39	440.90 (70.79)	20149.20 (3235.05)	5.14	10.4
Tianjin	109	107960 (17334)	30.98	290.90 (46.71)	15250.20 (2448.49)	4.12	5.9
Hebei	746	40255 (6463)	36.89	210.20 (33.75)	7517.00 (1206.89)	4.61	5.0
Shanxi	522	34919 (5606)	33.09	230.30 (36.98)	8045.50 (1291.74)	5.00	5.8
Inner Mongolia	807	71101 (11416)	36.45	218.00 (35.00)	8146.50 (1307.96)	5.33	6.5
Liaoning	543	65354 (10493)	36.01	259.40 (41.65)	8604.90 (1381.56)	6.09	6.0
Jilin	386	51086 (8202)	36.73	240.30 (38.58)	8406.90 (1349.77)	5.25	5.8
Heilongjiang	310	39462 (6336)	35.98	241.20 (38.73)	8285.70 (1330.31)	5.58	5.6
Shanghai	71	103796 (16665)	21.75	316.00 (50.74)	16084.60 (2582.46)	5.08	7.0
Jiangsu	855	87995 (14128)	27.02	235.50 (37.81)	10060.30 (1615.23)	5.19	6.1
Zhejiang	749	77644 (12466)	29.49	225.50 (36.21)	10578.00 (1698.35)	4.92	7.3
Anhui	893	35997 (5779)	29.82	203.20 (32.62)	6809.20 (1093.25)	4.35	4.6
Fujian	495	67966 (10912)	26.11	209.10 (33.57)	8173.50 (1312.30)	4.51	5.5
Jiangxi	846	36724 (5896)	27.54	213.00 (34.20)	6956.00 (1116.82)	4.33	4.6
Shandong	1569	64168 (10302)	31.95	229.40 (36.83)	8589.70 (1379.12)	5.27	6.3
Henan	1330	39123 (6281)	35.16	164.40 (26.40)	6874.40 (1103.72)	5.16	5.5
Hubei	519	50654 (8133)	33.52	214.30 (34.41)	7823.00 (1256.02)	5.86	6.3
Hunan	804	42754 (6864)	33.72	253.60 (40.72)	6980.10 (1120.69)	5.85	5.5
Guangdong	896	67503 (10838)	26.04	216.40 (34.74)	9982.20 (1602.69)	4.02	5.7
Guangxi	519	35190 (5650)	26.64	173.20 (27.81)	7154.60 (1148.71)	4.47	5.7

Chongqing	244	52321 (8400)	28.27	266.10 (42.72)	7481.80 (1201.24)	5.85	5.5
Sichuan	1542	36775 (5904)	29.67	219.20 (35.19)	7091.20 (1138.53)	5.96	5.8
Guizhou	174	29847 (4792)	23.39	228.90 (36.75)	5387.70 (865.02)	5.57	5.3
Yunnan	1061	28806 (4625)	30.24	179.70 (28.85)	5849.50 (939.17)	5.01	4.8
Shannxi	611	47626 (7647)	32.80	211.50 (33.96)	6604.30 (1060.35)	5.59	7.0
Gansu	475	26165 (4201)	29.87	169.70 (27.25)	5447.10 (874.56)	4.91	5.0
Qinghai	152	41252 (6623)	23.90	181.10 (29.08)	7980.20 (1281.26)	5.87	6.0
Xinjiang	95	40036 (6428)	24.94	208.40 (33.46)	6318.80 (1014.51)	6.37	6.9
Total	17370	49992 (8026)	29.27	233.90 (37.55)	8268.10 (1327.48)	5.11	5.8
Maximum	1569	107960 (17334)	36.89	440.90 (70.79)	20149.20 (3235.05)	6.37	10.4
Minimum	47	26165 (4201)	17.39	164.40 (26.40)	5387.70 (865.02)	4.02	4.6
Range ratio	33.38	4.13	2.12	2.68	3.74	1.58	2.26

Note: CNY – Chinese Yuan; OOP – out-of-pocket; THE – total health expenditure. Range ratio is the ratio of maximum to the minimum.

Source: China Statistical Yearbook of Health and Family Planning (2016).

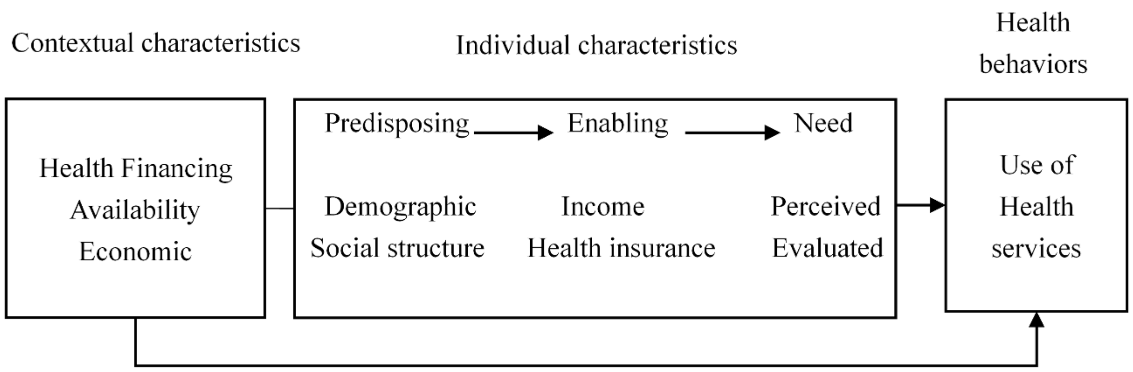

Figure S1 Analysis framework of health care utilization including health system and individual characteristics
